# Toxicity of Consecutive Treatments Combining Synthetic and Organic Miticides to Nurse Bees of *Apis mellifera*

**DOI:** 10.3390/insects16070657

**Published:** 2025-06-24

**Authors:** HeeJin Kim, Euijin You, JooHeon Cha, Si Hyeock Lee, Young Ho Kim

**Affiliations:** 1 Department of Ecological Science, Kyungpook National University, Sangju 37224, Gyeongbuk, Republic of Korea; maybe8541@naver.com; 2 Research Institute of Invertebrate Vector, Kyungpook National University, Sangju 37224, Gyeongbuk, Republic of Korea; yej8415@naver.com; 3 Department of Vector Entomology, Kyungpook National University, Sangju 37224, Gyeongbuk, Republic of Korea; 19cjh95@gmail.com; 4 Department of Agricultural Biotechnology, Seoul National University, Seoul 08826, Republic of Korea; shlee22@snu.ac.kr

**Keywords:** *Apis mellifera*, nurse bee, synthetic miticide, organic miticide, toxicity, *Varroa destructor*

## Abstract

*Varroa destructor*, an ectoparasitic mite of honey bees, is a major contributor to global colony declines. To manage infestations, beekeepers frequently apply both synthetic and organic miticides—sometimes in combination. While much attention has been given to foragers’ pesticide exposure, nurse bees are also at risk due to direct miticide contact and prolonged exposure to residuals in hive matrices. Despite this, little is known about the effects of sequential miticide applications on nurse bees. In this study, we evaluated the toxicities of single and pairwise consecutive applications of three synthetic miticides (fluvalinate, coumaphos, and amitraz) and two organic miticides (formic acid and oxalic acid) at field-realistic concentrations. Miticides were topically applied to nurse bees with 24 h between consecutive treatments. Synthetic miticides caused minimal mortality, while both organic miticides—especially formic acid—significantly reduced survivorship. Consecutive treatments generally produced mortality rates comparable to that of the corresponding organic miticide alone, indicating that observed toxicities were primarily driven by the organic agents. Notably, the fluvalinate–formic acid combination induced no significant adverse effects. These findings highlight the need for caution when using miticides in combination and support the development of safer application strategies to protect vulnerable in-hive bee populations..

## Simple Summary

Ectoparasitic mites pose serious threats to honey bee health and contribute to global colony losses. To manage mites, beekeepers commonly use both synthetic and organic miticides, sometimes in succession. While many studies focus on forager bees, nurse bees—who stay inside the hive—are also at risk due to direct contact and prolonged exposure to residual chemicals. This study investigated the toxic effects of single and consecutive applications of three synthetic (fluvalinate, coumaphos, and amitraz) and two organic (formic acid and oxalic acid) miticides, at field-realistic concentrations, on nurse bees. The results showed that synthetic miticides caused minimal acute mortality under the conditions tested, while organic miticides—especially formic acid—significantly reduced survival. In most cases, consecutive treatments did not worsen toxicity beyond that of the corresponding organic miticide alone, suggesting that the organic agents were the primary drivers of mortality. Overall, this study highlights the potential risks of combining miticides and underscores the need for safer application strategies to protect vulnerable in-hive bees like nurse bees.

## 1. Introduction

The western honey bee, *Apis mellifera*, is a key pollinator essential for both agricultural productivity and the maintenance of natural ecosystems. In addition, honey bees contribute significantly to beekeeping industries by producing valuable products, such as honey, propolis, and royal jelly. Despite their importance, global honey bee populations have been declining in recent years. Previous studies have reported that over 20% of honey bee colonies disappeared in the United States between 2018 and 2019 and in European countries between 2019 and 2020 [1,2]. This is a similar problem in the Republic of Korea, where severe colony losses were repeatedly reported during overwintering in 2021 and 2022 [3]. Various factors have been implicated in honey bee colony collapse, including infestations by ectoparasites, the spread of diseases, and abnormal climate conditions [3,4,5]. Among these, *Varroa destructor*, an ectoparasitic mite, is widely recognized as a major threat to honey bee health. Studies have demonstrated that *V. destructor* infection weakens the immune system, increases the susceptibility to viral infections, and inhibits the development of the honey bees, leading to colony collapse [6,7,8,9].

To control *V. destructor*, beekeepers worldwide employ various synthetic miticides, such as tau-fluvalinate (Flu), coumaphos (Cou), and amitraz (Ami), as well as organic miticides, including formic acid (FA) and oxalic acid (OA) [10,11]. Flu, Cou, and Ami exert neurotoxic effects on *V. destructor* by targeting voltage-gated sodium channels, acetylcholinesterase, and octopamine β receptors, respectively, while having relatively low toxicity to honey bees [12,13]. Among the organic miticides, FA acts by inhibiting cytochrome c oxidase [14], whereas the mode of action of OA remains unclear.

Despite their efficacy, the improper application of miticides can have detrimental effects on individual honey bees and entire colonies [3,15]. For example, Flu exposure has been shown to increase honey bees’ susceptibility to deformed wing virus [16], disrupt foraging behaviors [17,18], and impair queen reproductive capacity [19]. Similarly, Cou has been reported to reduce queen fertility and interfere with trophallaxis among workers [20,21]. Furthermore, the misuse of Ami has been associated with larval cell death and the inhibition of cuticle protease activity in worker bees [22,23].

Organic miticides can also cause significant harm if misapplied. Exposure to FA has been linked to cell death, respiratory interference, queen loss, and brood reductions [23,24,25,26,27]. Similarly, excessive OA use has been reported to incur cell death, reduced lifespans, and brood removal [7,26,28]. These findings underscore the risks associated with improper miticide usage in apiculture.

Recently, the emergence of *V. destructor* populations resistant to synthetic miticides, including Flu, Cou, and Ami, has been reported worldwide [5,29,30]. In the Republic of Korea, Flu-resistant *V. destructor* populations have rapidly increased since 2021 [31]. The widespread resistance of mites to synthetic miticides has made mite control increasingly difficult and has contributed to further honey bee losses. In response, many beekeepers have, without adequate knowledge of resistance dynamics, resorted to increasing miticide applications. Furthermore, as synthetic miticides alone are no longer sufficient for mite control, the simultaneous use of synthetic and organic miticides has become a common practice.

The continuous application of miticides leads to their accumulation in hive matrices, such as pollen and wax [10,32]. According to previous studies, when Checkmite^®^ strips containing Cou were used in colonies, the concentration of Cou significantly increased in wax [33,34]. In addition, colonies treated with commercial Ami products exhibited high Ami concentrations in their wax and bee bread when compared to untreated colonies [35]. Furthermore, Murcia-Morales et al. [36] reported that when miticides were administered to colonies, each miticide was detected in honey bees and colonies at high concentrations and remained present for weeks. In addition to bee products, various miticides were detected from living or dead honey bees, including adult bees and brood [32,37,38,39]. Calatayud-Vernich et al. [40] found miticide-contaminated honey bee carcasses in collapsed colonies. Together, these studies indicate that nurse bees are continuously exposed to synthetic miticides that persist inside the hive after application.

Furthermore, given that organic miticides are often introduced into hives where synthetic miticide residues persist and considering that both types of miticides are sometimes applied simultaneously—primarily exposing nurse bees rather than foragers—it is crucial to investigate their combined toxic effects on nurse bees. Therefore, in this study, we assessed the individual toxicity of commonly used synthetic (Flu, Cou, and Ami) and organic (OA and FA) miticides at field-realistic concentrations [41] to nurse bees. Then, we evaluated the synergistic toxicity resulting from sequential treatments involving synthetic and organic miticides, comparing the effects of single and combined treatments to identify miticide combinations that elicit unexpected toxic interactions in nurse bees.

## 2. Materials and Methods

### 2.1. Honey Bee Samples

Western honey bee nurse bees, identified based on their cell-cleaning and brood-care behaviors [42], were collected from the experimental apiary of Kyungpook National University in Sangju-si, Gyeongsanbuk-do, Republic of Korea. To enrich nurse bees, we collected individuals exclusively from the central brood frames during peak nursing hours (09:00–14:00), when brood care behaviors are most actively expressed. This practical approach, which has been widely adopted in previous studies [42], allows for non-lethal selection of nurse bees based on temporal task allocation and behavioral cues. To prevent unintended miticide exposure, no synthetic or organic miticides had ever been applied to the experimental colonies. Instead, the powdered sugar method was used for *V. destructor* control [43]. The collected nurse bees were maintained in an incubator (JSBI-250C, JS Research Inc., Gongju-si, Republic of Korea) at 32 °C under dark conditions and were provided with a 50% sucrose solution ad libitum.

### 2.2. Determination of the Experimental Miticide Concentrations

Analytical standard-grade synthetic miticides, including Flu, Cou, and Ami, were purchased from Sigma Aldrich (Saint Louis, MO, USA). Pure organic miticides, including FA and OA, were obtained from the National Institute of Agricultural Sciences of the Rural Development Administration (RDA, Jeonju-si, Republic of Korea).

To determine experimental concentrations reflective of field applications, we referred to previous studies [44,45,46,47] and the manufacturers’ manuals of commercial products registered with the Animal and Plant Quarantine Agency (APQA, Gimcheon-si, Republic of Korea) and the Environmental Protection Agency (EPA, Washington, DC, USA) (Table 1). For Flu, we referenced Apistan^®^ (Vita Bee Health Ltd., Basingstoke, UK), a product containing Flu as the active ingredient, since commercial products commonly used in Korea, such as Hongsabang^®^ (Shanxi Hengda Leiao Bio-Technology Co., Ltd.; Yuncheng, China) and Manpu Gold^®^ (Shanxi Weipeng Pharmaceutical Co., Ltd.; Yuncheng, China), have been less extensively studied. According to previous studies [44,46], Apistan^®^ strips release approximately 0.125 μg of Flu per day when applied to a honey bee colony following the manufacturer’s instructions. For Cou and Ami, we considered commercial products applied to honey bee colonies via spraying. The undiluted solutions of Coumaking^®^ (Daehan Nupharm, Hwasung-si, Republic of Korea) and Couma-H^®^ (Beesen Bio, Geumsan-gun, Republic of Korea) contain 32 g of Cou, and Soksal-Gold Solution^®^ (Shanxi Weipeng Pharmaceutical Co., Ltd.) and Dr+ Bee (Biobee, Hwaseong-si, Republic of Korea) contain 125 g of Ami. However, when diluted according to the manufacturers’ guidelines, the final concentrations of Cou and Ami in the prepared solutions are 0.62 μg/μL and 0.125 μg/μL, respectively. For organic miticides, the experimental concentration of FA was based on a previous study [47], which estimated that honey bees could be exposed to approximately 3 μL of FA per bee over 24 h when 60% FA was applied to a honey bee colony. For OA, since no standardized application method has been established in Korea, we referenced the concentration used in the dribble method, as indicated on product labels registered with the EPA.

### 2.3. Bioassay

To assess the toxicity of sequential miticide treatments, three synthetic miticides (Flu, Cou, and Ami) and two organic miticides (OA and FA) were applied to nurse bees at field-realistic concentrations. Synthetic and then organic miticides were administered sequentially, with a 24 h interval between treatments. Considering that certain miticides, such as Flu, FA, and OA, are applied through evaporation or fumigation, it was essential to ensure exposure to all body tissues. Given that the surface area of a honey bee is approximately 3 cm^2^ [48], 1 μL of each prepared miticide solution was topically applied to three body regions on each nurse bee: the head, thorax, and abdomen.

**Figure 1:**
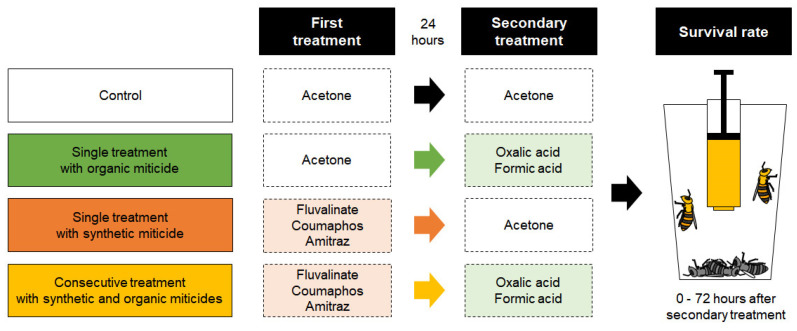
An overview of the experimental treatment procedure. Each group was administered with acetone and/or miticide as described in the left or right dotted boxes during first or secondary treatment, respectively, with a one-day interval. Organic miticides were categorized in light green, dotted box, whereas synthetic miticides were grouped in light orange, dotted box.

In the first treatment, nurse bees received 1 μL of either acetone (control) or a synthetic miticide in acetone at a specific concentration: 41.67 ppm for Flu, 626.67 ppm for Cou, and 125 ppm for Ami. Following treatment, the honey bees were maintained at 32 °C in darkness for 24 h in an incubator (JS Research Inc.) with ad libitum access to a 50% (*w*/*v*) sucrose solution. After 24 h, the second treatment was administered to the surviving nurse bees. Organic miticides were applied using the same method, with concentrations of 600,000 ppm for FA and 35,000 ppm for OA (Table 1). The treated nurse bees were then maintained under the same conditions used in the first 24 h (Figure 1).

The experimental design included sequential treatment groups in which nurse bees were treated with a synthetic miticide followed by an organic miticide. In the single-treatment groups, only one miticide type was applied, and pure acetone was used in place of the unused miticide. Thus, in the synthetic miticide single-treatment groups, nurse bees were treated with the synthetic miticide followed by acetone, while in the organic miticide single-treatment groups, acetone was applied first, followed by the organic miticide. The control group received acetone in both treatments.

Survival rates were recorded 0, 3, 6, 9, 12, 24, 36, 48, 60, and 72 h after the second miticide treatment. Honey bees were considered dead if they exhibited no movement upon stimulation, after which their carcasses were removed from the cages. All experiments were conducted in triplicate, with each experimental replicate performed at least three times.

### 2.4. Data Analysis

All survival rates from both single and sequential treatments were analyzed using SPSS 27 for Windows (IBM, Armonk, NY, USA). Kaplan–Meier survival analyses using log-rank test pairwise over strata were performed to assess overall survival differences among treatment groups. Survival rates at each time point were calculated as means ± standard errors (SEs) and compared using independent *t*-tests to determine statistical significance. Additionally, lethal time (LT) values, including LT_5_, LT_10_, and LT_30_, were estimated by extrapolation using Excel (Office 365, Microsoft, Redmond, WA, USA) following the method described by Mesa et al. [49], with slight modifications.

## 3. Results

### 3.1. Single Treatments with Synthetic Miticides

**Figure 2:**
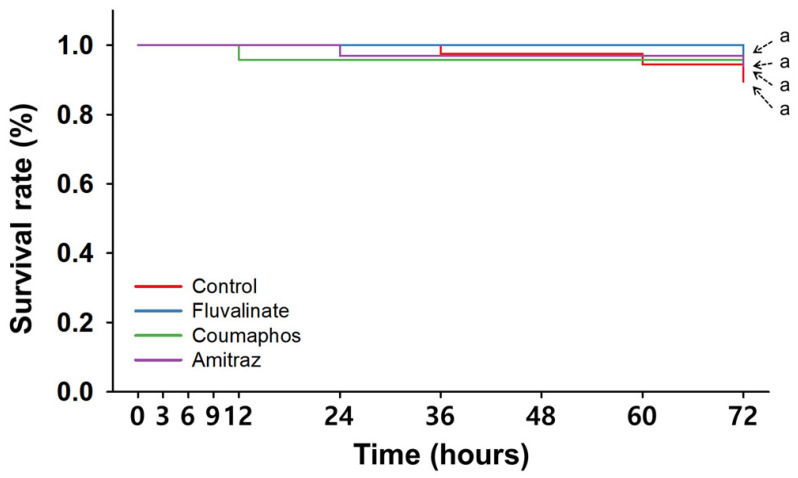
Time-dependent survival rate of nurse bees treated with synthetic miticides. Overall survival curve analyzed by Kaplan–Meier survival analysis with Log-rank test. Statistically significant differences (*p* < 0.05) among miticide treatments are indicated by different letters.

When evaluating the toxicity of single treatments with synthetic miticides at field concentrations, we found that the survival trends of nurse bees treated with Flu, Cou, and Ami were statistically similar to those of the control group across the entire 72 h post-treatment (hpt) period (*p* > 0.05) (Table S1 and Figure 2).

Based on the time-dependent data, the survival rate of the Flu group (100.0 ± 0.0%) was identical to that of the control group (100.0 ± 0.0%) at 24 hpt, while nurse bees exposed to Ami and Cou exhibited slightly lower but comparable survival rates of 96.9 ± 3.1% and 95.8 ± 4.2%, respectively (Table 2). At 48 hpt, survival rates remained high, with rates of 100.0 ± 0.0%, 95.8 ± 4.2%, 96.9 ± 3.1%, and 97.5 ± 2.5% recorded for the Flu, Cou, Ami, and control groups, respectively. Although the survival rates of nurse bees receiving the three synthetic miticides treatments were not statistically different at 72 hpt, nurse bees exposed to the miticides exhibited slightly higher survival than those of the control group, with Flu resulting in the highest (96.9 ± 3.1%), followed by Cou (95.8 ± 4.2%), Ami (93.8 ± 3.6%), and then the control (89.4 ± 4.1%). When attempting to calculate LT values, the LT_5_, LT_10_, and LT_30_ values for all treatment groups could not be reliably estimated because survival rates remained above 90% (Table 3).

Taken together, when exposed to single synthetic miticides at field-realistic concentrations, while slight variations in survival rates were observed among treatment groups at each time point, the overall survival rates of nurse bees were not significantly different from those of nurse bees in the control group over the 72 h observation period.

### 3.2. Single Treatments with Organic Miticides

**Figure 3:**
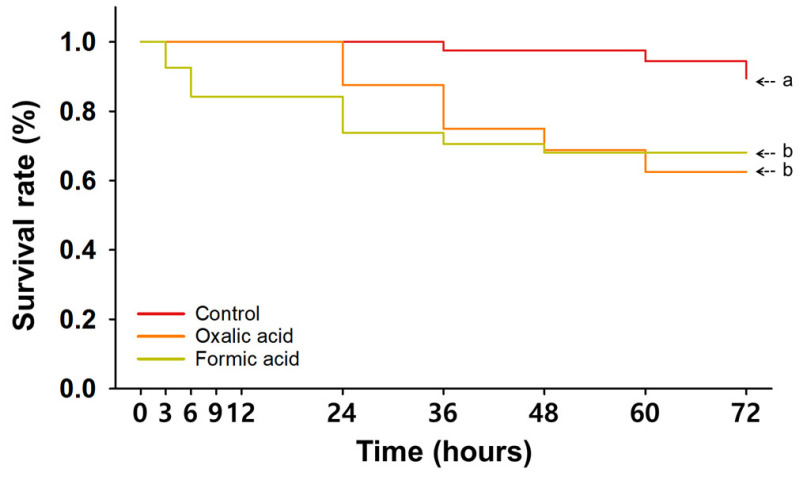
Time-dependent survival rate of nurse bees treated with organic miticides. Overall survival curve analyzed by Kaplan–Meier survival analysis with Log-rank test. Statistically significant differences (*p* < 0.05) among miticide treatments are indicated by different letters.

After single treatments with either OA or FA, the trends in nurse bee survival over time were significantly reduced when compared to the control group (*p* < 0.05); however, no statistical difference could be identified between the two organic miticide treatments (*p* > 0.05) (Table S2 and Figure 3). Notably, FA-treated nurse bees exhibited a more rapid decline in survival than those treated with OA within the 24 hpt (Figure 3).

When comparing survival at each observation period (Table 2), the OA-treated group only differed significantly from the control group at 60 hpt. In contrast, the FA treatment significantly reduced survival in the period from 36 to 60 hpt (Table 2). At 24 h after the second treatment, both organic miticides slightly increased mortality compared to the control; however, the survival rates of the OA (87.5 ± 12.5%, *p* > 0.05) and FA (73.8 ± 8.9%, *p* > 0.05) groups were not statistically different from that of control group (Table 2). At 48 hpt, OA-treated nurse bees exhibited a slightly lower survival rate of 68.8 ± 18.8%, which did not significantly differ from that of nurse bees in the control group (97.5 ± 2.5%) (*p* > 0.05). However, the FA treatment significantly reduced survival rates to 68.1 ± 8.4% (*p* < 0.05) after 48 hpt. By 72 hpt, survival rates were highest in the control group (89.4 ± 4.1%), followed by FA (68.1 ± 8.4%) and then OA (62.5 ± 12.5%), with no statistically significant difference between the two organic miticide treatments and the control (*p* > 0.05).

Table 3 presents the LT values for the OA and FA single-treatment groups. For OA, the LT_5_, LT_10_, and LT_30_ values were 16.0, 22.5, and 44.1 h, respectively. In contrast, FA exhibited a faster toxic response, with LT_5_, LT_10_, and LT_30_ values of 1.5, 4.9, and 29.4 h, respectively, indicating more rapid toxicity than OA.

In summary, FA and OA treatments significantly decreased overall survival rates (Figure 3), although survival did not differ significantly from the control group at certain time points. Additionally, FA produced a more rapid onset of toxic effects in nurse bees than OA (Table 3).

### 3.3. Consecutive Treatments with Synthetic and Organic Miticides

**Figure 4:**
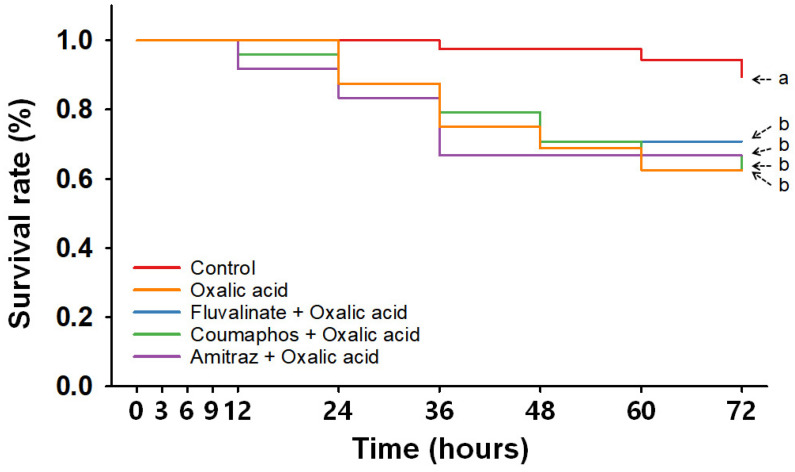
Time-dependent survival rate of nurse bees consecutively treated with synthetic miticides and oxalic acid. Overall survival curve analyzed by Kaplan–Meier survival analysis with Log-rank test. Statistically significant differences (*p* < 0.05) among miticide treatments are indicated by different letters.

To assess the toxicity of consecutive miticide treatments, nurse bees were exposed to synthetic and then organic miticides, with a 24 h interval between applications. Based on overall survival trends (Figure 4), nurse bees in the OA-combined treatment groups exhibited similar mortality patterns regardless of whether they had received a synthetic miticide treatment or the compound therein (*p* > 0.05) (Table S3 and Figure 4). However, all OA-treated groups experienced significantly higher mortality than the control group (*p* < 0.05) (Table S3).

When survival rates between the control group and OA-combined groups were compared at each time point (Table 2), treatment with Flu + OA produced nonsignificant differences across all time points except 36 and 48 hpt, whereas the Cou + OA and Ami + OA treatments induced significantly lower survivorship starting at 48 and 36 hpt, respectively. Specifically, at 24 hpt, the survival rate of the control group was 100.0 ± 0.0%, while it was slightly reduced in the Flu + OA (83.3 ± 8.3%, *p* > 0.05), Cou + OA (87.5 ± 7.2%, *p* > 0.05), and Ami + OA (83.3 ± 8.3%, *p* > 0.05) groups. In contrast, by 48 hpt, survival rates in all OA-combined groups were significantly lower than those of the control group (97.5 ± 2.5%), with survival rates recorded at 70.8 ± 11.0%, 70.8 ± 4.2%, and 66.7 ± 4.2% for the Flu + OA, Cou + OA, and Ami + OA groups (*p* < 0.05), respectively. By 72 hpt, survival rates in the Cou + OA and Ami + OA groups remained significantly lower than those in the control group (*p* < 0.05), but those in the Flu + OA and single OA treatment groups did not (*p* > 0.05). After 72 hpt, control nurse bees had the highest survival, (89.4 ± 4.1%), followed by those of the Flu + OA (70.8 ± 11.0%), Ami + OA (66.7 ± 4.2%), and then the Cou + OA (62.5 ± 7.2%) and OA-only (62.5 ± 12.5%) groups (Table 2).

**Figure 5:**
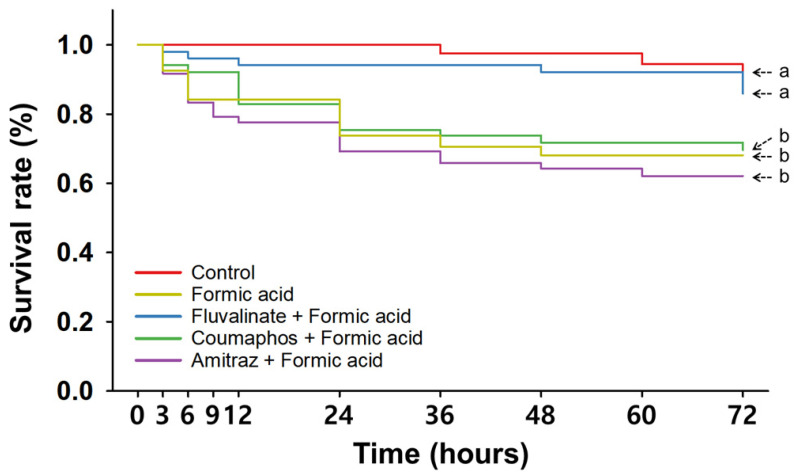
Time-dependent survival rate of nurse bees consecutively treated with synthetic miticides and formic acid. Overall survival curve analyzed by Kaplan–Meier survival analysis with Log-rank test. Statistically significant differences (*p* < 0.05) among miticide treatments are indicated by different letters.

In the consecutive treatments of synthetic miticides and FA (Figure 5 and Table S4), the overall survival trends of the Cou + FA and Ami + FA groups were significantly lower than that of control group (*p* < 0.05); however, the Flu + FA treatment did not produce a significantly different survival trend compared with the untreated control group (*p* > 0.05). Compared to the other treatments, the survival trend of nurse bees after the FA single treatment was statistically different from those of the control and Flu + FA groups (*p* < 0.05), but similar to those of the Cou + FA and Ami + FA groups (*p* > 0.05).

In comparisons at different time points (Table 2), Flu + FA exhibited no significant difference in survival rates at any recorded time, while Ami + FA showed significantly lower survival at 24 hpt and thereafter. Furthermore, the Cou + FA and FA-only groups had significantly lower survival rates than the control group from 24 to 60 hpt and 36 to 60 hpt, respectively. At 24 hpt, survival in the Flu + FA group (94.2 ± 2.6%) exhibited a slight, nonsignificant decline (*p* > 0.05), whereas survival in the Cou + FA (75.4 ± 5.3%, *p* < 0.05) and Ami + FA (69.2 ± 5.8%, *p* < 0.05) groups was significantly lower than in the control group (100.0 ± 0.0%). Though survival in the FA single-treatment group (73.8 ± 8.9%) showed a clear reduction at 24 hpt, it was not significantly different from that in the control (*p* > 0.05). At 48 hpt, the Flu + FA group (92.1 ± 2.5%, *p* > 0.05) exhibited a survival rate similar to that of the control (97.5 ± 2.5%), but the Cou + FA (71.7 ± 7.4%) and Ami + FA (64.2 ± 7.5%) treatments significantly reduced survival (*p* < 0.05). In contrast to the 24 hpt results, the FA-only group exhibited significantly lower survival at 48 hpt (68.1 ± 8.4%, *p* < 0.05). By 72 hpt, survival rates among most OA-treated groups were not significantly different from that of the control group (*p* > 0.05 for Flu + FA, Cou + FA, and FA-only), but the Ami + FA group remained significantly lower (*p* < 0.05). At 72 hpt, the control group boasted the highest survival (89.4 ± 4.1%), followed by the Flu + FA (85.9 ± 3.9%), Cou + FA (69.6 ± 7.5%), and FA-only (68.1 ± 8.4%) groups, with the Ami + FA treatment resulting in the lowest (62.1 ± 7.9%).

When LT values of OA-combined groups were calculated using extrapolation (Table 3), LT_5_ values of the Flu + OA, Cou + OA, and Ami + OA groups were 12.6, 13.2, and 9.9 h, respectively, and LT_10_ values were 18.7, 20.0, and 15.8 h, respectively. The LT_30_ values of nurse bees consecutively treated with synthetic miticides and OA were estimated to be between 39.0 and 43.1 h. When FA was combined with synthetic miticides, LT values varied greatly depending on the specific chemical used. The LT_5_ and LT_10_ values of Flu + FA-treated nurse bees were calculated to be 15.7 h and 70.5 h, respectively, but the LT_30_ could not be calculated because survival rates remained above 90% after treatment. In contrast, the LT_5_, LT_10_, and LT_30_ values of the Cou + FA treatment were 3.5, 7.4, and 56.5 h, respectively, and those of the Ami + FA treatment were 1.2, 3.7, and 24.0 h, respectively.

In summary, after consecutive treatments with synthetic miticides and then organic miticides, survivorship of nurse bees was generally significantly lower than that of nurse bees in the control group, except for the combination of Flu and FA (Figure 5). Interestingly, similar survival trends were usually observed between each single organic miticide treatment and the consecutive treatments involving the same organic miticide, suggesting that organic miticides may be particularly toxic to nurse bees, but no synergistic effects were produced by synthetic miticides (Table 2).

## 4. Discussion

This study evaluated the individual and combined toxicities of synthetic (Flu, Cou, and Ami) and organic (FA and OA) miticides on nurse bees of *A. mellifera* using field-realistic concentrations and exposure protocols that reflect common apicultural practices. Our findings demonstrated that while synthetic miticides exerted minimal acute toxicity, organic miticides—particularly FA—caused substantial mortality. Furthermore, the toxicity observed in consecutive treatments was largely attributable to the organic miticides rather than any synergistic effects with synthetic agents.

Aligning with some studies [7,15], nurse bees exhibited high survival rates following single exposures to Flu, Cou, and Ami at field concentrations, with no significant differences from the control group (Table S1 and Figure 2). However, these results differ from other studies reporting higher toxicities at comparable or even lower doses [22,50,51]. Such discrepancies may be explained by methodological differences, including differences in the physiological age of the treated honey bees, application methods, and observation durations. For instance, the use of nurse bees—rather than foragers or mixed-age workers—may have influenced susceptibility, given the demonstrated age-dependent variation in detoxification enzyme expression and immune function in honey bees [52,53].

In contrast, both FA and OA significantly decreased survival (Table S2 and Figure 3), with FA inducing a more rapid onset. The estimated lethal times (LT_5_, LT_10_, and LT_30_) for FA were markedly shorter than for OA, indicating a more acute toxic response (Table 3). These findings align with previous reports that high doses or the improper application of organic miticides can cause physiological stress, cellular damage, and behavioral abnormalities [26,27]. The topical application to the head, thorax, and abdomen used in our study likely facilitated rapid systemic absorption, potentially exacerbating neurotoxic effects. Notably, the application of acids to head tissues can induce local acidosis in neural tissues, leading to protein denaturation and neuronal damage [24,54].

In the sequential treatment assays, combined applications of synthetic and organic miticides generally did not produce effects beyond those observed with organic miticides alone, and thus, did not demonstrate additive or synergistic effects. Survival rates in most combined groups were not statistically different from the corresponding single organic miticide treatment (Figure 4 and Figure 5 and Tables S3 and S4), suggesting that the organic agents were primarily responsible for the observed toxicity. This is further supported by the lack of significant differences in LT values between single and combined exposures involving organic miticides. An exception was the combination of Flu and FA. After treatment with this combination, nurse bees maintained survival rates comparable to the control group bees’ survival rates throughout the observation period. This unexpected result may be due to the two compounds producing opposing physiological effects; Flu has been reported to increase respiratory rates [55,56], while FA is known to inhibit mitochondrial respiration [24].

The potential antagonism between these compounds warrants further investigation and highlights the importance of metabolic interactions between miticides. Interestingly, our findings contradict those of Johnson et al. [57], who reported reduced toxicity when combining Ami and OA, suggesting potential protective interactions. In our study, the Ami + OA treatment induced a relatively strong toxicity, much greater than that of Ami alone (Table 2). This discrepancy may arise from differences in treatment order; Johnson et al. [57] applied OA before applying Ami, which may have induced hydrolysis and the degradation of Ami [58], whereas our protocol applied Ami first. Thus, the persistence of Ami under acidic conditions may explain the enhanced toxicity in our sequential treatment.

The accumulation of miticide residues in hive matrices such as wax, pollen, and bee bread, as reported in previous studies [32,40], represents a persistent source of exposure for nurse bees. Residues of Flu, Cou, and Ami degradation products have been detected at considerable concentrations, sometimes exceeding 200 ppm. Given that nurse bees are in prolonged contact with hive materials, they are particularly vulnerable to chronic exposure and potential sublethal effects.

Taken together, our data demonstrate that organic miticides—particularly FA—pose a greater acute risk to nurse bees than their synthetic counterparts when applied at field-relevant concentrations. While synthetic miticides did not exacerbate organic miticide toxicity under the conditions tested, repeated or improper application in field settings may lead to cumulative effects. The variability in survival rates between treatment combinations underscores the important influences of application order, compound interactions, and the physiological state of the honey bees. Although our study focused on acute mortality, we recognize that sublethal effects of miticides—including those on glandular development, gut microbiota, and larval care behaviors—may also significantly impair nurse bee function and colony health. Future investigations incorporating physiological and behavioral endpoints are warranted to fully characterize miticide-related risks.

In conclusion, our findings underscore the necessity for caution when using organic miticides, especially in combination treatments. While synthetic miticides exhibited minimal acute toxicities at field concentrations, the inclusion of organic miticides significantly compromised nurse bee survival. Miticide application strategies should be reevaluated with the aim of minimizing collateral harm to critical in-hive populations such as nurse bees, whose loss may disrupt brood care and colony development. Future research should investigate the chronic and sublethal effects of miticide combinations, as well as their physiological mechanisms of action. In addition, the 72 h observational period employed in this study was selected to capture acute mortality trends based on previous miticide toxicity studies. However, we acknowledge that extended observation periods (e.g., ≥10 days) may reveal delayed or cumulative effects not detected within this timeframe. Further long-term studies are warranted to fully assess the chronic impacts of miticide exposure on nurse bee survival.

## Figures and Tables

**Table 1:** Information on the miticides tested in this study and their field-realistic concentrations.

Chemical		Product (A.I. %) ^a^	Estimated Exposure Concentration	References
Synthetic miticide	Fluvalinate	Apistan (824 mg/strip)	41.67 ppm ^b^	[44,45,46]
	Coumaphos	Coumaking (3.2%)	626.67 ppm ^c^	Product label registered at the APQA
		Couma-H (3.2%)	626.67 ppm ^c^	
	Amitraz	Soksal-gold Solution (12.5%)	125 ppm ^c^	
		Dr+ Bee Solution (12.5%)	125 ppm ^c^	
Organic miticide	Formic acid	Handmade solution	600,000 ppm ^b^	[47]
	Oxalic acid	Handmade solution	35,000 ppm ^c^	Product label registered at the EPA

^a^ Active ingredient (%) in the commercial products; ^b^ estimated field-realistic exposure concentration based on the previous studies; ^c^ Recommended concentration (ppm) when the application solution is made following the guidelines registered with the Animal and Plant Quarantine Agency (APQA, Republic of Korea) or Environmental Protection Agency (EPA, USA).

**Table 2:** Survival rates (arithmetic mean ± standard error) of nurse bees at multiple time points after single and consecutive treatments with miticides.

Time (hpt ^a^)	Control	Single Treatment					Consecutive Treatment					
		Fluvalinate	Coumaphos	Amitraz	Oxalic Acid	Formic Acid	Fluvalinate + Oxalic Acid	Coumaphos + Oxalic Acid	Amitraz + Oxalic Acid	Fluvalinate + Formic Acid	Coumaphos + Formic Acid	Amitraz + Formic Acid
3	100 ± 0.0	100 ± 0.0	100 ± 0.0	100 ± 0.0	100 ± 0.0	92.5 ± 5.0	100 ± 0.0	100 ± 0.0	100 ± 0.0	97.9 ± 2.1	94.2 ± 4.2	91.7 ± 5.3
6	100 ± 0.0	100 ± 0.0	100 ± 0.0	100 ± 0.0	100 ± 0.0	84.2 ± 7.3	100 ± 0.0	100 ± 0.0	100 ± 0.0	96.1 ± 2.5	92.1 ± 4.1	83.3 ± 7.7
9	100 ± 0.0	100 ± 0.0	100 ± 0.0	100 ± 0.0	100 ± 0.0	84.2 ± 7.3	100 ± 0.0	100 ± 0.0	100 ± 0.0	96.1 ± 2.5	92.1 ± 4.1	79.2 ± 9.5
12	100 ± 0.0	100 ± 0.0	95.8 ± 4.2	100 ± 0.0	100 ± 0.0	84.2 ± 7.3	100 ± 0.0	95.8 ± 4.2	91.7 ± 4.2	94.2 ± 2.6	82.9 ± 6.5	77.5 ± 8.9
24	100 ± 0.0	100 ± 0.0	95.8 ± 4.2	96.9 ± 3.1	87.5 ± 12.5	73.8 ± 8.9	83.3 ± 8.3	87.5 ± 7.2	83.3 ± 8.3	94.2 ± 2.6	75.4 ± 5.3 **	69.2 ± 5.8 ***
36	97.5 ± 2.5	100 ± 0.0	95.8 ± 4.2	96.9 ± 3.1	75 ± 12.5	70.6 ± 10.6 *	75.0 ± 7.2 *	79.2 ± 11.0	66.7 ± 4.2 ***	94.2 ± 2.6	73.8 ± 6.4 *	65.8 ± 6.6 **
48	97.5 ± 2.5	100 ± 0.0	95.8 ± 4.2	96.9 ± 3.1	68.8 ± 18.8	68.1 ± 8.4 *	70.8 ± 11.0 *	70.8 ± 4.2 ***	66.7 ± 4.2 ***	92.1 ± 2.5	71.7 ± 7.4 *	64.2 ± 7.5 **
60	94.4 ± 3.3	100 ± 0.0	95.8 ± 4.2	96.9 ± 3.1	62.5 ± 12.5 *	68.1 ± 8.4 *	70.8 ± 11.0	66.7 ± 4.2 ***	66.7 ± 4.2 ***	92.1 ± 2.5	71.7 ± 7.4 *	62.1 ± 7.9 *
72	89.4 ± 4.1	96.9 ± 3.1	95.8 ± 4.2	93.8 ± 3.6	62.5 ± 12.5	68.1 ± 8.4	70.8 ± 11.0	62.5 ± 7.2 *	66.7 ± 4.2 *	85.9 ± 3.9	69.6 ± 7.5	62.1 ± 7.9 *

^a^ hours post-treatment (hpt); survival rates of each treatment group were statistically compared to the control group at the same time point. Statistical significance is denoted using asterisks: *, *p* < 0.05; **, *p* < 0.01; and ***, *p* < 0.001.

**Table 3:** Estimated median lethal times (LTs) of the five single and six consecutive miticide treatments against nurse honey bees.

Treatment		Estimated Lethal Times (h)		
		LT_5_	LT_10_	LT_30_
Single treatment	Fluvalinate	>72 *	>72 *	>72 *
	Coumaphos	>72 *	>72 *	>72 *
	Amitraz	>72 *	>72 *	>72 *
	Oxalic acid	16.04	22.46	44.09
	Formic acid	1.49	4.92	29.41
Consecutive treatment	Fluvalinate + Oxalic acid	12.62	18.67	38.99
	Coumaphos + Oxalic acid	13.18	19.98	42.74
	Amitraz + Oxalic acid	9.93	15.77	43.11
	Fluvalinate + Formic acid	15.71	70.54	>72 *
	Coumaphos + Formic acid	3.45	7.35	56.50
	Amitraz + Formic acid	1.15	3.74	24.04

* Honey bee groups treated with the miticide or miticide combination are estimated to reach the corresponding LT mortality level after 72 hpt.

## Data Availability

The original contributions presented in this study are included in the article/Supplementary Materials. Further inquiries can be directed to the corresponding author.

## Supplementary Materials

The following supporting information can be downloaded at: https://www.mdpi.com/article/10.3390/insects16070657/s1, Table S1: *p* value from Log-Rank test of Kaplan-Meier survival analysis in single treatment groups with synthetic miticide.; Table S2: *p* value from Log-Rank test of Kaplan-Meier survival analysis in single treatment groups with organic miticide.; Table S3: *p* value from Log-Rank test of Kaplan-Meier survival analysis in consecutive treatment groups with synthetic miticide and oxalic acid.; Table S4: *p* value from Log-Rank test of Kaplan-Meier survival analysis in consecutive treatment groups with synthetic miticide and formic acid.
