# Supplementary material for: Toxicity of Consecutive Treatments Combining Synthetic and Organic Miticides to Nurse Bees of Apis mellifera
Source: Insects. 2025 Jun 24;16(7):657. doi: 10.3390/insects16070657 (PMC12294837; doi:10.3390/insects16070657)
Supplement: Supplementary file 1 [file insects-16-00657-s001.zip › insects-3682465-supplementary.pdf]

Supplementary Information

Table S1. *P*-values of log-rank tests from a Kaplan–Meier survival analysis of nurse bees subjected to single treatments with synthetic miticides.

	Control	Fluvalinate	Coumaphos	Amitraz
Control	-	0.228	0.393	0.536
Fluvalinate	0.228	-	0.826	0.549
Coumaphos	0.393	0.826	-	0.750
Amitraz	0.536	0.549	0.750	-

Table S2. *P*-values of log-rank tests from a Kaplan–Meier survival analysis of nurse bees subjected to single treatments with organic miticides.

	Control	Oxalic acid	Formic acid
Control	-	0.011	0.022
Oxalic acid	0.011	-	0.823
Formic acid	0.022	0.823	-

Table S3. *P*-values from Kaplan–Meier log-rank tests comparing the survival curves of nurse bees following consecutive treatments with one of three synthetic miticides and then oxalic acid.

	Control	Oxalic acid	Fluvalinate + Oxalic acid	Coumaphos + Oxalic acid	Amitraz + Oxalic acid
Control	-	0.011	0.044	0.007	0.017
Oxalic acid	0.011	-	0.652	0.988	0.905
Fluvalinate + Oxalic acid	0.044	0.652	-	0.608	0.720
Coumaphos + Oxalic acid	0.007	0.988	0.608	-	0.887
Amitraz + Oxalic acid	0.017	0.905	0.720	0.887	-

Table S4. *P*-values from Kaplan–Meier log-rank tests comparing the survival curves of nurse bees following consecutive treatments with one of three synthetic miticides and then formic acid.

	Control	Formic acid	Fluvalinate + Formic acid	Coumaphos + Formic acid	Amitraz + Formic acid
Control	-	0.022	0.631	0.022	0.003
Formic acid	0.022	-	0.044	0.912	0.527
Fluvalinate + Formic acid	0.631	0.044	-	0.042	0.005
Coumaphos + Formic acid	0.022	0.912	0.042	-	0.386
Amitraz + Formic acid	0.003	0.527	0.005	0.386	-
